# Examining GP online consultation in a primary care setting in east midlands, UK

**DOI:** 10.1186/s12913-021-07039-2

**Published:** 2021-09-30

**Authors:** Dewy Nijhof, Andy Ingram, Rebecca Ochieng, Emma-Jane Roberts, Barnaby Poulton, Bertha Ochieng

**Affiliations:** 1 Faculty of Health and Life Sciences, De Montfort University, Leicester, UK; 2 Xcelerate Health Outcomes, London, UK; 3 Optimum Patient Care, Cambridge, UK; 4 Digital Health Evaluation Services, London, UK

**Keywords:** Digital health technology, E-consultation, Online consultation, Asynchronous care, Synchronous care, Online GP consultation, Primary care, Remote consultation

## Abstract

**Background:**

Increasing pressure threatens to overwhelm primary care services, affecting the quality of care and their role as gatekeepers to specialised care services. This study investigated healthcare users’ acceptability of – and the effectiveness of – an e-consultation system in primary care services.

**Methods:**

Seven GP practices in East-Midlands, all of whom use online consultation system participated in the study, with a retrospective review being undertaken of 189 electronic patients’ records (age range of 18–76 years) over 5 months. The focus was on the electronic records of patients who accessed the service for five different conditions identified as presenting common conditions seen by the GPs practices. Statistical analysis was done using SPSS to perform an exploratory data analysis and descriptive statistics.

**Results:**

The results showed a positive reception of the online consultation platform, with an average satisfaction score of 4.15 (most likely to recommend score = 5). Given the nature of the conditions, 47.6% of patients had experienced a previous episode of the health condition they were seeking consultation for, and a total of 72% had existing comorbidities. Follow-up activity occurred for 87.3% of patients, 66.1% of which included at least one follow-up visit for the same condition as the initial online consultation.

**Conclusion:**

The results suggest that online consultation is convenient for patients, and it also has the potential to relieve pressure placed on primary care services. Although a number of challenges were identified, such as patient verification, this study gives insight into – and enhances our understanding of – the use of online GP consultations.

## Introduction

Increasing demand for primary care services has put pressure on general practices (GPs), affecting their role as gatekeepers to specialised care services [1] as well as affecting patient satisfaction and management of workload [2, 3]. New approaches, therefore, must handle primary care demand in a flexible, efficient, and cost-effective way [1]. A promising approach is online GP consultation (e-consultation), defined as a telemedicine consultation in which the consulting expert is the clinician and the remote client is a patient [4]. E-consultation can occur synchronously (i.e. real-time using video calls or phone calls) or asynchronously (i.e. texting, email, apps) using a store-and-forward method [5].

Although online mediums were initially used amongst clinicians [6, 7], patients reportedly reached out to GPs via email as early as 1998 [6]. Despite growing interest [8], user rates remained low [9], and research on the use of these mediums only started to pick up in 2007 [10]. Usage then increased, with e-consultation being implemented in health care systems and regional networks across America, Canada, Northern England, Ireland, the Netherlands, and the Ukraine [11, 12].

There is a general positivity towards e-consultations [9, 13], with trials suggesting improved outcomes for health issues such as diabetes and hypertension [5]. GPs report confidence in decision-making via these mediums when it concerned existing conditions, monitoring, or uncomplicated concerns, but not when the presenting condition was new [14]. The perceived impact on clinicians’ workloads varied, with some feeling it required more time [11] while others felt that it saved time [14, 15] or did not differ at all [16, 17]. Where follow-ups were required, more information was needed. Still, GPs remain hesitant about e-consultation [18, 19], perhaps because its use in the UK is associated with unregulated and unrecorded e-consultations by private practices [20, 21]. Additionally, concerns remain about its security/confidentiality and its accessibility to people who struggle with technology, such as the ageing population [5, 22].

Several e-consultation models have been implemented in the UK that are supported by the NHS, such as WebGP (renamed eConsult) and askmyGP. These models have shown an improved access of care and a reduction in appointment time [23]. However, rate of use was low [16, 24], which is thought to be due to a lack of patient and staff engagement, insufficient support, and a lack of protocols [16]. Usage mirrored typical surgery hours and involved mostly administrative requests in addition to some medication requests [22, 24]. Use of e-consultation in the UK is still in its infancy [12], prompting the NHS to provide funds to promote the use and development of this technology [25].

This study aimed to explore user experiences and usage patterns of an online consultation system with regard to selected acute and chronic conditions. By exploring usage of the service, this study sought to extend results to perceived acceptability and effectiveness of the online consultation system in terms of treatment and online consultation outcomes for selected conditions. To do this, retrospective patient records for selected conditions were explored.

## Methods

This study used data that was routinely collected by Docly online consultation and the NHS electronic patient record systems (SystmOne) from 189 patients. A total of 1154 patients used the e-consultation services during the time of this study, 225 of which used the services for the selected indications, agreeing to take part in the study. Informed consent was obtained from each patient and all methods were carried out in accordance with relevant guidelines and regulations.

Patients participating in this study were signed up at one of seven GP practices in East-Midlands that use Docly services. Inclusion criteria consisted of seeking consultation for tonsillitis, a cough, urinary tract infection, acne, vaginosis, sinusitis, and eczema within the five-month study period (November 2019 – March 2020). These indications were chosen as they are the common conditions seen by the GPs practices and that can be self-managed, given the right advice. They represent both acute and chronic conditions and have set patient management guidelines or disease scoring mechanisms. Exclusion criteria consisted of not receiving any advice via Docly or receiving advice via Docly for a condition other than the selected indication. In addition, participants had to be 18 years or older at the time of diagnosis and capable of consenting.

The online consultation process included patients logging into the online service, selecting their health problem, and then filling in a guided questionnaire about their symptoms. Decision support algorithms recommended the most appropriate outcome to the clinician based on the answers of the questionnaire, before either providing immediate advice to the patient or before a further assessment was made by a GP. Health problems that required more complex care were referred to specialist care services. Emergency situations were flagged by the smart algorithms, with patients receiving advice to contact urgent care services.

Data on the e-consultation was extracted from the Docly system according to the inclusion criteria. The second stage of the data extraction included developing a Data Capture Form (DCF), according to the inclusion/exclusion criteria, in order to extract data from the participating practices’ SystmOne. The DCF included information on the patient’s demographics, health condition, use of Docly, and Docly satisfaction scores. Data analysis included a data-cleaning process to remove incomplete and inaccurate data. SPSS was used to analyse the data on demographics, patient satisfaction score, number of consultations, previous episodes of the initial indication, and follow-up activity, as well as the distribution of age and gender for all these factors. This included frequencies and descriptive of the abovementioned variables split by indication, gender, age and type of consultations. Thirdly, data visualisation was carried out using RStudio.

## Results

### Demographics

**Fig. 1:**
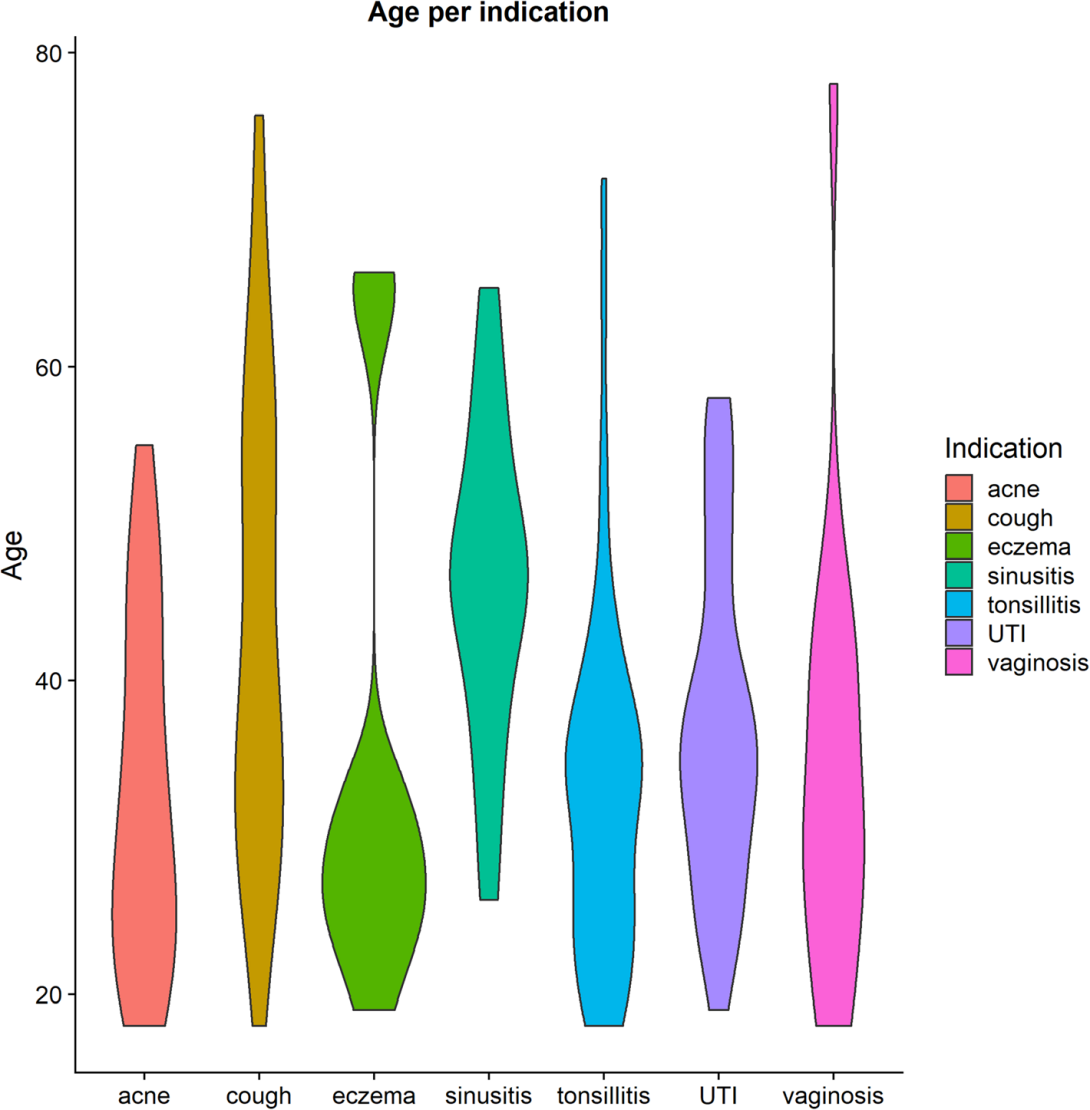
Age distribution per indication

The 189 participants had an average age of 37.2 years (SD = 12.26, range 18–78) and consisted of mostly females (see Table 1). Figure 1 shows the distribution of age per indication. All health conditions follow similar age distributions with the exception of eczema and sinusitis. Table 2 shows the demographics for the e-consultation and follow-ups as well as the medication that was prescribed in each of these consultations for the selected indication. Age and gender distributions in each type of consultation are similar to that of the overall sample.

**Table 1:** Distributions of total sample and gender per age range

Age range	Total sample	Men	Women
< 19 years	11 (5.8%)	1 (9.1%)	10 (90.1%)
20–29 years	49 (25.9%)	8 (16.3%)	41 (83.7%)
30–39 years	62 (32.9%)	14 (22.6%)	48 (77.4%)
40–49 years	33 (17.4%)	6 (18.2%)	27 (81.8%)
50–59 years	23 (12.2%)	7 (30.4%)	16 (69.6%)
60–69 years	7 (3.7%)	2 (28.6%)	5 (71.4%)
> 70 years	4 (2.1%)	3 (75.0%)	1 (25.0%)

**Table 2:** The percentage of medication prescribed for each visit according to the patient reported medical condition

	Online consultation	Follow-up 1	Follow-up 2	Follow-up 3	Follow-up 4	Follow-up 5
Patients	189	165	124	92	69	47
Gender distribution	41 men	34 males	24 males	17 males	13 males	8 males
	148 females	131 females	100 females	75 females	56 females	39 females
Age	*M* = 37.2	*M* = 36.75	*M* = 37.38	*M* = 36.86	*M* = 36.35	*M* = 35.17
	(*SD* = 12.26)	(*SD* = 13.47)	(*SD* = 14.55)	(*SD* = 14.30)	(*SD* = 14.50)	(*SD* = 14.55)
Medication	34 (18%)	55 (33.3%)	10 (8.1%)	11 (12%)	2 (2.9%)	1 (2.1%)

### Medication prescription

Medication was mostly prescribed in follow-up 1 and, throughout this study, 47 different medications were prescribed. These were categorised into antibiotics, dermal creams, respiratory medication, allergy medication, birth control, and other (i.e. anti-fungal, anti-inflammatory, and pain relief). The most prescribed medication was the antibiotic Nitrofurantoin, for UTIs. This medication was prescribed mostly in the e-consultation or at the first follow-up. In the context of this study, the second most prescribed medication was Amoxicillin, for tonsillitis and cough symptoms. The third most prescribed medication was Phenoxymethylpenicillin, for tonsillitis.

### Comorbidities, previous episodes, and follow-up activity

**Fig. 2:**
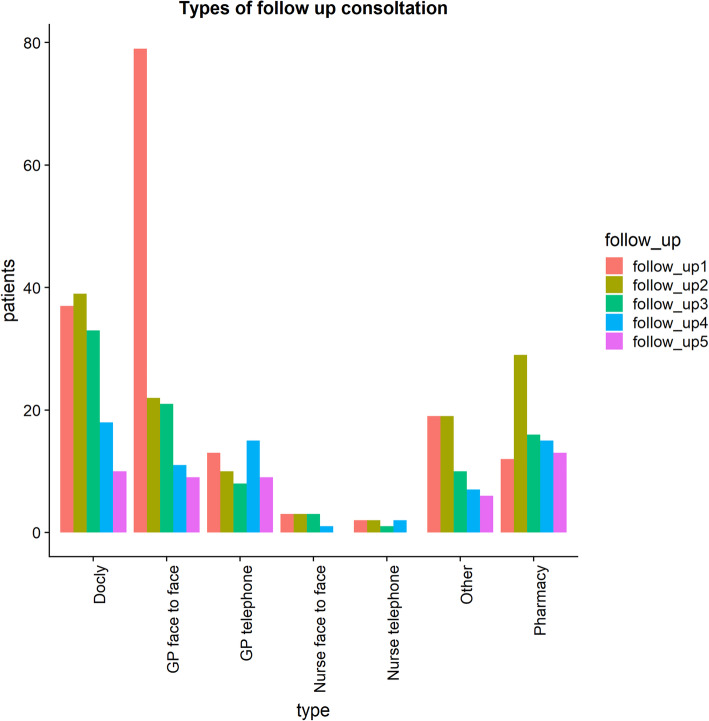
The number of patients per consultation and follow-up. Pharmacy in this graph represents pharmacy prescriptions requested via Docly

Table 3 shows the distribution of men and women split by indication. Again, women form the majority of patients. In addition, Table 3 shows the amount of comorbidities split by indication. A total of 136 patients (72%) had at least one comorbidity. The most common comorbidities were mental health issues and “other”. The category “other” contains comorbidities that were not present in a large enough number of patients to justify their own category, including conditions such as anaemia, vitamin deficiencies, polycystic ovary syndrome, and migraines. Almost half (47.6%) of all patients had had a previous episode of their selected condition. Most patients had experienced one-two previous episodes, while patients with a cough, UTI, and sinusitis had experienced five or more previous episodes of their indication (see Table 3). Follow-up activity occurred for 165 (87.3%) patients, 109 (66.1%) of which included at least one follow-up visit for the same indication as the e-consultation. Tonsillitis, cough, vaginosis, and sinusitis had the most patients seeking more than five follow-up consultations (see Table 3). Figure 2 shows the number of patients who initially accessed the online consultation and the percentage of the follow-up. The most sought-after type of follow-up appointment was a face-to-face with the GP, namely in follow-up 1. Of these 79 patients seeking a GP face-to-face follow-up, 71 were advised to do so in their online consultation. The second most sought-after type of consultation was via Docly online, whether it was for a specific health condition or pharmacy requests.

**Table 3:** The distribution of gender per indication, comorbidities, previous episodes of the indication and the number of follow-ups]

	Total	Men	Women	0 co-morbidities	1 co-morbidity	2 co-morbidities	3 ≥ co-morbidities	0 previous episodes	1 previous episode	2 previous episodes	3 previous episodes	4 previous episodes	5 previous episodes	5 ≥ previous episodes	0 follow ups	1 follow up	2 follow ups	3 follow ups	4 follow ups	5 follow ups	5 ≥ follow ups
Tonsillitis	51 (27.9%)	17 (33.3%)	34 (66.7%)	16 (31.4%)	16 (31.4%)	7 (13.7%)	12 (23.5%)	31 (60.8%)	15 (29.4%)	3 (5.9%)	1 (2.0%)	1 (2.0%)	–	–	6 (11.8%)	10 (19.6%)	7 (13.7%)	5 (9.8%)	8 (15,7%)	3 (5.9%)	12 (23.5%)
Cough	43 (22.4%)	16 (37.2%)	27 (62.8%)	10 (23.3%)	9 (20.9%)	9 (20.9%)	15 (34.9%)	17 (39.5%)	9 (20.9%)	8 (18.6%)	5 (11.6%)	1 (2.3%)	1 (2.3%)	2 (4.7%)	2 (4,7%)	11 (25,6%)	8 (18,6%)	6 (14.0%)	4 (9.3%)	2 (4.7%)	10 (23.3%)
UTI	33 (17.4%)	–	33 (100.0%)	11 (33.3%)	13 (39,4%)	1 (3.0%)	8 (24.2%)	16 (48.5%)	7 (21.2%)	4 (12.1%)	2 (6.1%)	2 (6.1%)	1 (3.0%)	1 (3.0%)	3 (9.1%)	8 (24,2%)	6 (18,2%)	5 (15,2%)	3 (9,1%)	2 (6,1%)	6 (18,3%)
Acne	20 (10.5%)	2 (10.0%)	18 (90.0%)	9 (45.0%)	3 (15.0%)	2 (10.0%)	6 (30.0%)	11 (55.0%)	5 (25.0%)	4 (20%)	–	–	–	–	4 (20.0%)	5 (25.0%)	6 (30.0%)	2 (10.0%)	–	2 (10.0%)	1 (5.0%)
Vaginosis	18 (9.6%)	–	18 (100.0%)	5 (27.8%)	4 (22.2%)	5 (27.8%)	4 (22.2%)	14 (77.8%)	4 (22.2%)	–	–	–	–	–	2 (11,1%)	3 (16,7%)	3 (16,7%)	2 (11,1%)	2 (11,1%)	1 (5,6%)	5 (27,8%)
Sinusitis	14 (6.9%)	3 (21.4%)	11 (78.6%)	1 (7.1%)	3 (21.4%)	3 (21.4%)	7 (50.0%)	7 (50.0%)	5 (35.7%)	1 (7.1%)	–	–	–	1 (7.1%)	4 (28,6%)	2 (14,3%)	1 (7,1%)	1 (7,1%)	3 (21,4%)	–	3 (21,4%)
Eczema	10 (5.4%)	3 (30.0%)	7 (70.0%)	1 (10.0%)	2 (20.0%)	2 (20.0%)	5 (50.0%)	3 (30.0%)	3 (30.0%)	2 (20.0%)	2 (20.0%)	–	–	–	3 (30%)	1 (10.0%)	2 (20.0%)	1 (10.0%)	2 (20.0%)	–	1 (10.0%)

### Recommended outcomes

**Fig. 3:**
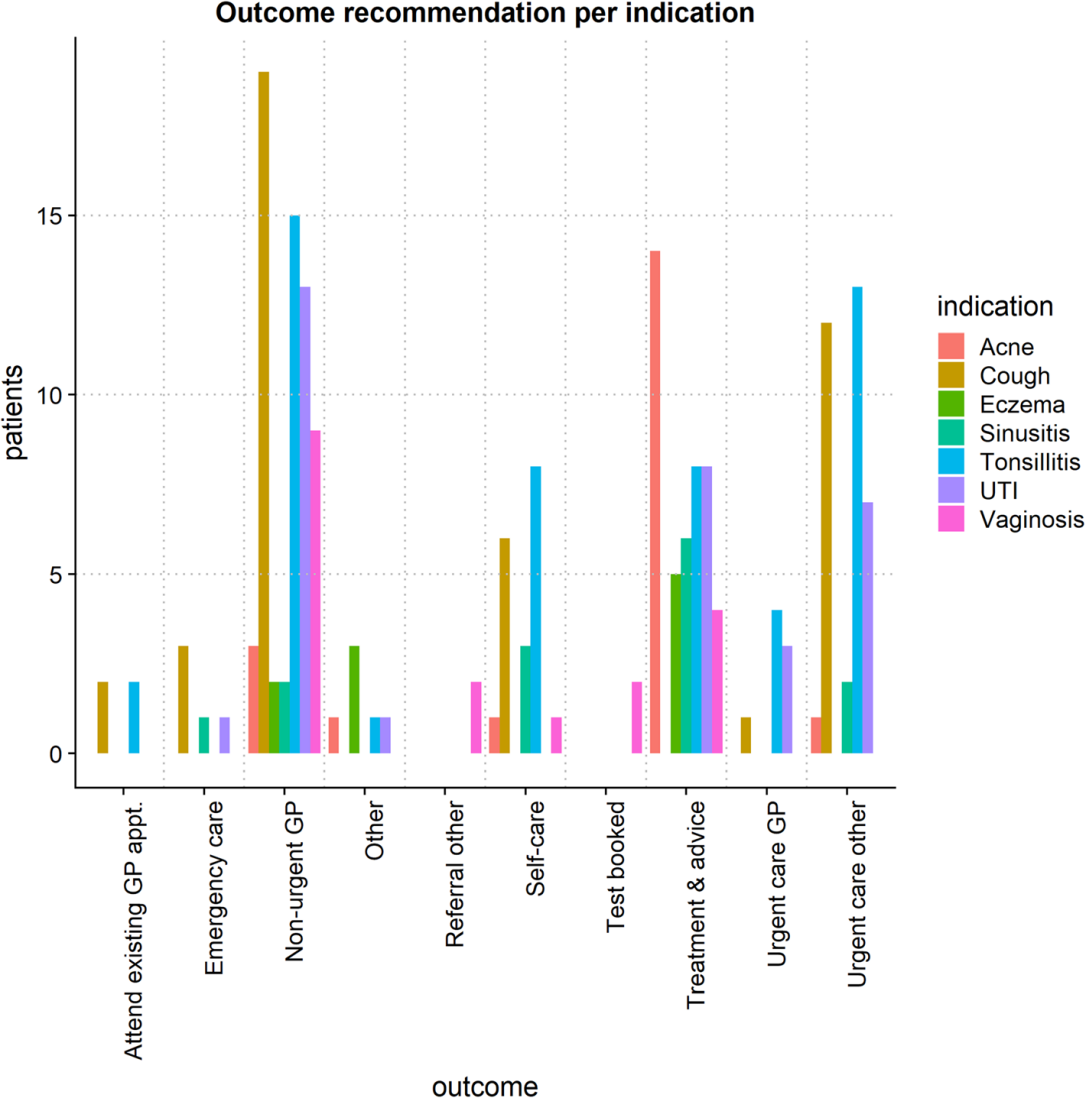
Number of patients, indication and outcome type following the online consultation

Ten categories of recommended outcomes resulted from the e-consultation: emergency care, urgent care (other), urgent GP appointment, non-urgent GP appointment, attending an existing GP appointment, treatment and advice, test booked (e.g. swabs), referral other (e.g. sexual health clinic), self-care, and other (e.g. no response from patient). Figure 3 shows the number of patients per outcome type for all conditions, patients with a cough were mostly recommended either to book a non-urgent GP appointment or seek urgent care. Similarly, recommended outcomes for patients with tonsillitis differed greatly, with a non-urgent GP appointment slightly higher followed by urgent care and a similar number of patients being recommended for self-care or treatment and advice. Patients with UTIs were mostly recommended a non-urgent GP appointment, but smaller numbers of patients were recommended treatment and advice or urgent care. Patients seeking consultation for acne, eczema, or sinusitis were mostly recommended treatment and advice.

### Patient compliance to medical advice

In total, 75 (39.7%) patients were recommended to see a GP (urgent, non-urgent, and to attend previously booked GP appointments). Of the 8 (4.2%) patients recommended an urgent GP appointment, 7 patients followed through. A total of 67 (35.5%) patients were recommended to either book a non-urgent GP appointment or to attend a previously planned GP appointment, and 43 patients followed this advice. A total of 40 (21.2%) patients were recommended in their e-consultation to seek urgent/emergency care, but only 16 patients followed this advice. Thus, 25 patients sought urgent care during follow-up 1 of their own accord. Self-care advice was given to 19 (10.1%) patients, while 6 of these patients booked at least one follow-up consultation for the same condition either via e-consultation, GP, or emergency/urgent care. Of these patients that booked a follow-up consultation, only one was prescribed additional medication for their condition, suggesting that their condition could have worsened.

Of 189 patients, 78 (41.3%) patients responded to the satisfaction questionnaire about their experiences of using the online consultation, of which 4 (5.1%) responded with “I don’t know”. On a scale of 1–5, with 1 being the least likely to recommend and 5 being the most likely to recommend, the average satisfaction score was 4.15 (SD = 1.02).

## Discussion

This study explored the use and effectiveness of e-consultation in primary care services. Patients used the services almost equally for new and existing conditions as well as interchangeably with other primary care services, such as face-to-face GP consultations. Advice offered in the e-consultation seemed sufficient for 1/3 patients, whereas 2/3 needed at least one follow-up. Although this may indicate adherence to best practice guidelines and normal diagnostic flow (in which multiple consultations lead to the correct diagnosis), this may also reflect the patient’s inability to accurately articulate their signs and symptoms, or to classify their condition, which could lead to a gap in health advice and which could ultimately lead to follow-up calls or face-to-face appointments. This study provides a uniquely detailed description of how this e-consultation system has been used by patients alongside traditional primary care services.

### Demographics

The mean age of the total sample and individual conditions is similar to the mean age reported in previous online consultation systems [10, 16, 22, 24], with the exception of sinusitis. The age distribution for sinusitis is in line with the increased likelihood of sinusitis occurring in an ageing person [26], possibly due to physical changes in sinus areas, poorer blood circulation, and less efficient clearance of mucus from the nose, which comes with age [27]. Similarly, a flare-up at an older age (> 60) for eczema corresponds to physiological ageing, as the skin structure changes with age, increasing susceptibility to skin injuries and, concomitant with immune senescence, vulnerability to pathology [28, 29]. The wider age range again corresponds to literature showing that although acne is most common amongst teenagers and younger individuals, it is not a rare occurrence in adults [ 30]. In line with previous pilots of online consultation systems [10, 16, 22, 24], the majority of the patients in this study were female.

### Medication prescription

Medication was frequently prescribed in follow-up 1. It is possible that this was because not all prescribed medications are suitable for online consultation. For example, this may be the case for medications where weight, height, age, and the severity of the condition need to be taken into account. The most prescribed medications for the first follow-up consultation were: Phenoxymethylpenicillin, Amoxicillin, and Nitrofurantoin. According to the NICE guidelines, the prescription of these antibiotics is dependent on the presentation alongside other symptoms, comorbidities, and risk of complications [31].

### Comorbidities, previous episodes, and follow-up activity

In primary care, mental health issues frequently occur concurrently with medical conditions such as asthma, diabetes, obesity, and cardiovascular diseases [32]. This study did find a high prevalence of both asthma and mental health issues in the current sample, but no such correspondence with regards to diabetes, obesity, or cardiovascular diseases. The high prevalence of mental health issues in this sample is important to note, as mental health comorbidity may contribute to increased utilisation of health care services [33]. For example, anxiety sensitivity has been linked to anxiety focused on bodily sensations [34], and both anxiety-related symptoms and non-anxiety-related symptoms are strongly associated with hypochondriacal fears [35]. Also referred to as being ‘health anxious’, these patients are often dissatisfied or even frustrated with the care they receive, something that is shared by their physicians [36]. Moreover, there is an established link between epidemic outbreaks and pandemic-related health anxiety [37–39], and reports of COVID-19-specific health anxiety are starting to emerge [40]. In the current sample, it is not known when mental health diagnoses were established, thus it is not possible to link the usage of this online consultation medium to pandemic-related health anxiety. However, based on the literature on pandemic-related health anxiety it is not unreasonable to assume it has played a role in care-seeking behaviours of the current sample, however small that may be.

Almost half (47.6%) of all patients had experienced a previous episode of the indication they were seeking a consultation for. The majority of patients who had experienced a previous episode had only had one or two previous episodes. When looking at this number in the context of the selected indications, this is not surprising. Conditions such as acne [41] or eczema [42] are often chronic or persist for long periods of time. Similarly, UTIs are often recurrent [43], and vaginosis is deemed both persistent and recurrent [44]. Likewise, it is not uncommon to have recurrent episodes of chronic sinusitis [45] or tonsillitis [46]. Coughing is a common symptom that may indicate a wide variety of conditions, both acute and chronic. It is also the most common reason for a patient to seek medical help [47].

Overall, e-consultation comprised 44.7% of all follow-up consultations (see Fig. 3). A possible reason for this is that e-consultation could be used as a way to navigate the different access points of the primary care system. Previous studies on online consultation specifically found this type of “abuse of the system” [14, 22].

### Recommended outcomes

The most recommended outcome from the online consultation was for the patient to book a non-urgent GP appointment. This outcome was particularly high for patients presenting with a cough, tonsillitis, UTI, or vaginosis. For coughs and tonsillitis, it is likely that the throat needs to be examined closely in order to establish a diagnosis. Indeed, consultation forms reported that not every patient was able to take a clear picture of their throat.

The most common cause of acute cough (i.e. with a duration of less than two weeks) is a viral upper respiratory tract infection, which generally does not require assessment or therapy [47] and which may explain why non-urgent GP appointments are recommended instead of urgent care or urgent GP appointments. That being said, there is still a high number of patients with a cough who were recommended urgent care (other). This may indicate an adherence to NICE guidelines, where no antibiotic or immediate antibiotic treatment is recommended for a cough depending on the presentation alongside other symptoms, comorbidities, and risk of complications [31]. Similarly, the need for urgent care and treatment of tonsillitis is highly dependent on the severity of the symptoms and a general feeling of wellbeing, as well as patient-specific factors such as risk for complications [48], which may explain the high numbers of patients that are being recommended to book a non-urgent GP appointment, seek urgent care, self-care, or treatment and advice. Correspondingly, UTIs may be classified as uncomplicated or complicated depending on the patient’s individual risk of failing therapy [49]. Therefore, the finding of high numbers for both non-urgent GP and urgent care recommendations – as well as treatment and advice recommendations – may be due to individual risk.

### Online consultation: challenges

The present study used data obtained from Docly systems as well as SystmOne. Literature on online consultations notes a concern for patient identity verification [10], which is a main point for opposition to these systems [20, 21]. Indeed, during data extraction, two patients explicitly sought consultation for other individuals. These individuals have been excluded from the data analysis. However, while patients asking advice for other individuals is not uncommon even for face-to-face consultations, it is likely that the data used in this study would contain medical advice sought for individuals other than the listed patient. For e-consultations, it is critical that patients are made aware of the risks of seeking advice for individuals who are not registered with the GP practice. In such instances, the prescribing GP will not, for example, know of the patients’ health history, current medication, or any allergic reactions. Therefore, there is a risk of the GP not picking up red flags during such an online consultation, which could compromise the patient’s health.

All online consultations for the seven indications were submitted before the UK coronavirus lockdown measures were put in place, with the last consultation being submitted within the first week of March 2020. Follow-up activity was recorded up until 3 months after the initial online consultation, therefore partly or mostly taking place during the first UK lockdown. Follow-up activity does show a slight rise in telephone consultations accompanied by a large decrease in face-to-face consultations. This is consistent with stay-at-home measures and the NHS guidelines of turning to remote consultations unless face-to-face consultation is absolutely necessary [50]. Indeed, there has been an increase – albeit a slow one [51] – in the use of remote consultations, especially in telephone consultations [52], even for out-of-hours care [53].

### E-consultation: future consideration

The results from this study can substantially improve our understanding of how e-consultation systems are used by patients, which can then inform the implementation of these systems and the improvement of existing systems. For example, the use of this online consultation system by patients suggests that, in contrast to GPs [18], patients feel as confident in using e-consultation for both new and existing conditions – as is evident in almost half of the patients having had a previous episode and little over half of the patients having had no previous episode of their selected condition. In addition, data for the type of consultation sought for follow-ups suggest that patients switch between their use of the General Practice and online mediums, which possibly reflects the convenience or the exploration of access points to primary care. Moreover, the high referral to see a GP face-to-face in follow-up 1, along with the high number of prescriptions in follow-up 1 (Table 2), may suggest that some conditions are less suitable for online consultations.

The literature on online consultations notes a concern for patient identity verification [10], a notion shared by GPs and pharmacists who oppose online consultation [20, 21]. Indeed, during data extraction we identified two patients records who were seeking for medical advice for others (one in addition to seeking medical advice for themselves, the other only for another individual). Both cases were excluded from the study and urged by GPs to seek advice from the individual’s own physician.

### Limitation

The data cleaning process brought to light that patients occasionally experienced difficulty identifying the indication that best suited their symptoms. In addition, the focus was on a limited set of conditions that GPs commonly saw, and therefore, excluded the experience of patients whose symptoms were not part of the selected indication. Another critical limitation of the study was its reliance on existing electronic patient records. While this has highlighted the experiences of patients accessing online consultation, future studies could explore differences in ethnicity, GP’s perspectives of using online consultation, and the views of patients who do not access online consultation. Such data would inform and enhance the implementation of online consultation.

## Conclusion

This study explored the use of an online GP consultation system in remarkable detail, allowing for unique insight into how online consultations are used. Both the reported satisfaction and patterns of use suggest that online GP consultation is acceptable and effective, that patients found it convenient, and that it has the potential to relieve the amount of pressure put on traditional primary care services. Despite the high patient satisfaction rate, the results of this study highlight areas that need to be considered when implementing online consultations, such as concerns regarding patient verification and simplification of the user interface.

## Data Availability

The datasets used and/or analysed during the current study are available from the corresponding author on reasonable request.

## Abbreviations

GP: General Practitioner/General practices
UK: United Kingdom
NHS: National Health Services
DCF: Data Capture Form
UTI: Urinary Tract Infection
NICE: National Institute for Health and Care Excellence
